# Vaccination roll-out: a time to develop and maintain trust in science and health care

**DOI:** 10.3399/bjgp21X717629

**Published:** 2021-10-29

**Authors:** Orla T Muldoon, Daragh Bradshaw, Sarah Jay, Elaine L Kinsella, Paul Maher, Robert Murphy, Carol Taaffe, Patrick O’Donnell

**Affiliations:** Centre for Social Issues Research, University of Limerick, Limerick.; Centre for Social Issues Research, University of Limerick, Limerick.; Centre for Social Issues Research, University of Limerick, Limerick.; Centre for Social Issues Research, University of Limerick, Limerick.; Centre for Social Issues Research, University of Limerick, Limerick.; Research Services and Policy Unit, Department of Health, Dublin.; Research Services and Policy Unit, Department of Health, Dublin.; School of Medicine, University of Limerick, Limerick.

## INTRODUCTION

Many countries are facing a new phase of the pandemic where COVID-19 vaccine roll-out and uptake takes centre stage. Vaccine hesitancy poses a real challenge in pursuit of this goal. Indeed, the World Health Organization (WHO) listed vaccine hesitancy as one of the top 10 threats to global health.^1^ The need to understand and support uptake of COVID-19 vaccinations is now imperative. To achieve herd immunity, the virus transmission rate, R, and the performance of the vaccine must be taken into account.^2^ Given higher transmissibility of new variants, and an optimistic estimate of efficacy of .80, reducing the risk of vaccine recipients getting the disease by 80%, herd immunity may require entire populations to be immunised.^2^^,^^3^

## REVIEW FINDINGS

Reflecting the WHO’s concern,^1^ a recently published concise review^4^ highlighted that there is significant disparity in uptake rates across countries. Historically, Organisation for Economic Co-operation and Development (OECD) countries are those with the poorest vaccine uptake. Here we outline the latest empirical evidence on important individual- and group-level factors that influence COVID-19 vaccine intentions, and include specific evidence-based recommendations for GPs facilitating vaccination roll-out. Studies of adults’ vaccination intentions in OECD countries were systematically reviewed.^5^ Of the 31 eligible studies, eight (26%) were rated as high quality, 13 (42%) were rated as good quality, and 11 (35%) were rated as satisfactory. None of the studies were excluded from the review as the appraisal process evaluates reporting rather than conduct and content, which usefully informs findings and discussion.^6^

We looked at all quantitative surveys of nationally representative samples published in the 19 months to the end of July 2021 that asked responders about their planned vaccine intentions. Although survey and response formats varied, generally the proportion of responders who intended to vaccinate fell short of that required to achieve herd immunity in all 31 studies. Between 60% and 80% of those surveyed reported that they intended to ‘definitely’ or ‘possibly’ take the vaccine.^5^ This, along with the likely gap between intention to vaccinate and vaccination uptake,^7^ indicates that encouraging vaccination among those living in OECD countries remains a significant task.

An early rapid review examined studies published from June to October 2020.^8^ It suggested that intention to vaccinate declined as the pandemic wore on. These authors suggested that the appearance of safety concerns, waning public trust in governments, and misinformation might lie at the heart of this decline. One longitudinal study has now been published that appears to confirm this concern.^9^

In our review, as more studies have become available, people’s reasons for both their intention to accept or refuse the vaccine are becoming clearer. The factors most often identified as underlying willingness to vaccinate are: 1) vaccine efficacy and clinical safeguards in testing; 2) the return to family, social, and working life facilitated by health protection associated with vaccination; and 3) good vaccine literacy or knowledge.^10^^–^^12^ Frequently cited factors associated with unwillingness included concerns about side effects, conspiracy beliefs, and a belief that the vaccine development was rushed.^10^^,^^13^^,^^14^

These findings highlight the need for transparency about the science behind the vaccines and the importance of honest information about safety and efficacy, as well as the side effects, to help shore up trust-based willingness to vaccinate where it is found. When information is presented accessibly through traditional and new media channels, it has the potential to improve vaccine literacy and dispel the erroneous beliefs that drive unwillingness.^10^

In 26 of the 31 studies reviewed, demographic factors such as age, sex, and education were modelled as potential predictors of willingness to vaccinate. Group variations were prevalent and remarkably consistent: where sex, age, and education differences were apparent, older people (21/22 studies), males (22/28 studies), and those with higher levels of education (19/21 studies) reported greater intention to vaccinate. Similarly where it was examined, majority ethnic and racial (11/15 studies) groups and high-income (12/12 studies) groups often reported higher willingness to vaccinate. For historical reasons, groups such as males, older people, and the more educated majority ethnic groups may have fewer reasons to mistrust the system, scientists, and governments. For other groups — females, those most deprived, and the less educated — trust in science, medicine, government, and pharma is comparatively poorer.

This dearth of trust is not without justification. Marginalised groups have had their trust in science, government, and the healthcare system violated before and even during this pandemic.^15^ Age is also relevant. Lower perceived risk among the young is a factor here. In addition, economic deregulation since the 1980s has resulted in some questionable practices by healthcare and pharmaceutical providers. Millennials have learned to question the motives of scientists, governments, and even healthcare workers.^16^

Hoping to inform how intentions may be shaped, many of the studies reviewed sought to identify predictors of vaccine beliefs and behaviour. Knowledge was not a strong predictor: it was influential in only one of five models.^5^ In cross-national studies,^13^^,^^15^ differences in levels of trust in national governments was strongly associated with vaccine acceptance and intentions. Within countries, trust and confidence in science, government, and public health officials was predictive of vaccine intentions in 11 of the 12 studies. Importantly, the focus of all these studies is vaccine intentions, not vaccine uptake. Typically, a substantial gap between vaccination intentions and behaviour^7^ can erode vaccination uptake. Identifying factors that translate good intentions into vaccination uptake is an important area of future research.

## TRANSLATING THESE FINDINGS TO PRACTICE

Building trust in vaccination programmes among the hesitant is now an important task. There are groups that will need more encouragement and support to be vaccinated. For the most part, greater effort will be needed to persuade the young, females, and minority ethnic groups. Sensitive and targeted communication is essential for those at high risk of low uptake.^12^ Communication about the value of the vaccine roll-out needs to be nuanced to the concerns of those who, by virtue of their sex, age, and educational or ethnic group position, are least likely to be involved in decision-making processes. Those in less powerful groups tend to have a strong sense of the collective. It can be seen to arise from the solidarity of disempowered social status. This strong sense of collective values a relational orientation, over and above individual choice or autonomy.^17^ A message emphasising the value of the vaccine to ‘us all’, including family, social, and national group, is likely to be particularly potent in these communications.^18^

While public health campaigns can offer this nuanced information on the efficacy and safety offered to family and community by the vaccine, lack of trust may undermine the messages from government and health authorities among key target groups. It is important, therefore, to not only think about the message but also the messenger. GPs and other trusted healthcare providers with whom these groups have a pre-existing and trusting relationship have an important role. Healthcare liaison workers serving and working with groups at risk of low uptake are central opinion leaders to combating vaccine hesitancy. GPs and public health officials should ensure that these community-based personnel understand and are supportive of the vaccine roll-out and vaccination programme. Communication from GPs and healthcare workers embedded within communities, and who have established trusting relations over time, are far most likely to be effective than public health broadcasts.^19^

## VACCINE UPTAKE AND DIVERSE POPULATIONS

Vaccine uptake is a social and political issue as much as a medical one.^20^ Groups that occupy positions of power by virtue of their age, professional status, sex, or ethnicity often think that they understand the concerns of the less powerful in their care. We are not attuned to thinking of generations, sexes, and educational groups as distinct cultural groups. Yet they are just this. And though we may believe that we are working to the concerns of the many, often we are informed only by a few. Policy and practice that pays attention to diversity and is informed by populations as diverse as those we seek to vaccinate are those likely to reap the greatest reward.
